# Cerebellar Transcranial Direct Current Stimulation Effects on Saccade Adaptation

**DOI:** 10.1155/2015/968970

**Published:** 2015-03-02

**Authors:** Eric Avila, Jos N. van der Geest, Sandra Kengne Kamga, M. Claire Verhage, Opher Donchin, Maarten A. Frens

**Affiliations:** ^1^Department of Neuroscience, Erasmus MC, 3000 CA Rotterdam, Netherlands; ^2^Department of Biomedical Engineering, Ben Gurion University of the Negev, 84105 Beer-Sheva, Israel; ^3^Erasmus University College, 3011 HP Rotterdam, Netherlands

## Abstract

Saccade adaptation is a cerebellar-mediated type of motor learning in which the oculomotor system is exposed to repetitive errors. Different types of saccade adaptations are thought to involve distinct underlying cerebellar mechanisms. Transcranial direct current stimulation (tDCS) induces changes in neuronal excitability in a polarity-specific manner and offers a modulatory, noninvasive, functional insight into the learning aspects of different brain regions. We aimed to modulate the cerebellar influence on saccade gains during adaptation using tDCS. Subjects performed an inward (*n* = 10) or outward (*n* = 10) saccade adaptation experiment (25% intrasaccadic target step) while receiving 1.5 mA of anodal cerebellar tDCS delivered by a small contact electrode. Compared to sham stimulation, tDCS increased learning of saccadic inward adaptation but did not affect learning of outward adaptation. This may imply that plasticity mechanisms in the cerebellum are different between inward and outward adaptation. TDCS could have influenced specific cerebellar areas that contribute to inward but not outward adaptation. We conclude that tDCS can be used as a neuromodulatory technique to alter cerebellar oculomotor output, arguably by engaging wider cerebellar areas and increasing the available resources for learning.

## 1. Introduction

Saccades are performed in order to foveate targets of interest. These fast and brief eye movements cannot rely on online (visual) feedback since visual delays are longer than the movement itself. This means that, in order to maintain accurate eye movements, the motor commands for future saccades must be adjusted after each eye movement is completed. These plastic mechanisms are present to reduce or compensate motor errors due to either physiological or pathological behavior [1, 2]. Since McLaughlin (1967) described the “parametric adjustment,” known today as short-term saccade adaptation, his paradigm has been used as a way to assess learning and plasticity in the oculomotor system. This is done by asking a subject to make a saccade to a new position and while the saccade is in flight, the target moves (intrasaccadic step) causing a postsaccadic visual error [3–5]. When the subject is repeatedly exposed to the same error, the oculomotor system will gradually drive a change in the metrics of the eye movement over time, making the error smaller [6–15]. The error can induce saccade shortening (gain-down), when the intrasaccade step of the target is in the direction of the starting point of the saccade (inward adaptation), or saccade lengthening (gain-up) when the step is away from the starting point (outward adaptation). Human subjects adapt faster in response to inward adaptation than in response to outward adaptation stimuli [1], which poses the hypothesis that these two types of adaptation involve different neural mechanisms [16, 17].

The cerebellum plays a crucial role in saccadic error detection [18–20] and thus in saccade adaptation [2]. Evidence of the cerebellar involvement and its necessary integrity to oculomotor learning has been demonstrated as large lesions, focal inactivation, or pathological conditions of different areas of the cerebellum impair the ability to adapt saccades [21–25]. In addition, various loci in the cerebellum relate to inward and outward errors differently [18]. For instance, patients with vermal damage are partially capable of inward adaptation but lack outward adaptation [24]. Also, MRI-guided TMS on lateral hemispheres potentiates the postadaptation effects of outward adaptation and, in contrast, depresses gain-down adaptation [26].

Neuromodulatory techniques can be used to influence functional roles in various brain structures. Cerebellar output can be modulated with transcranial direct current stimulation (tDCS) with great specificity as shown by excitability changes after stimulation ranging from cognitive to motor skills [27–30]. In this study, we used anodal tDCS as a tool to noninvasively modulate cerebellar output and provide functional insight into the learning aspects during saccade adaptation.

## 2. Materials and Methods 

### 2.1. Participants

13 healthy subjects (one author: E. Avila, 12 naive subjects to tDCS, mean age of 22.4, range 19–29 years, 6 females), right-handed volunteers with no known history of neurological or psychiatric conditions, not taking chronic or acute medications or using drugs, with normal vision, were recruited. They all gave informed consent to participate in the experiment, which was approved by the local medical ethics committee and adhered to the Declaration of Helsinki. Ten subjects participated in the inward saccade adaptation experiment and ten in the outward saccade adaptation experiment. Seven subjects participated in both experiments.

### 2.2. Setup

Subjects were seated in a completely darkened room at 84 cm in front of a 21 in. computer screen. The screen was covered with a red filter to eliminate light reflections of the monitor and after images. Eye movements were recorded binocularly at 250 Hz by means of video-oculography (SR Research EyeLink II, Ontario, Canada) [31]. Head movements were restrained by a chin rest and monitored throughout the measurements to ensure head stability.

### 2.3. Task

**Figure 1:**
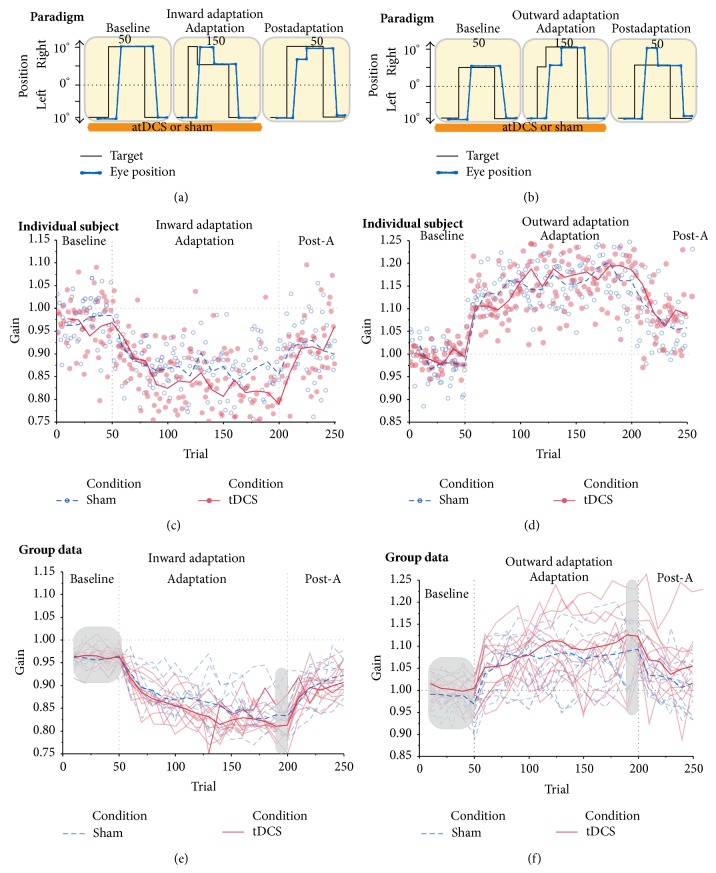
Experimental paradigms, single subject data, and population data. Panel (a) depicts inward adaptation where subjects performed an inward paradigm that consisted of 50 baseline trials of 20° saccades at intervals between 1.5 and 2 s, followed by 150 adaptation trials where the second target had an intrasaccadic step of 5°. Eye trace shows an overshoot at the beginning of the phase and the subject makes a corrective saccade to the target. Finally, 50 postadaptation trials presented in the same way as baseline trials. Anodal tDCS was delivered for 15 min at the start of the experiment or for 30 s in the sham condition. Panel (b) shows outward adaptation consisting in the same trial structure as inward, but here the subjects experienced baseline trials of 15° saccades and a forward jump of 5° (in the direction of the saccade). The middle row shows examples of adaptation for a single subject in the inward adaptation (gain-decrease, panel (c)) and outward adaptation (gain-increase, panel (d)) experiment for the two tDCS conditions. Lines on the top depict blocks composed of the median values of 10 trials. The bottom row shows group data for inward (panel (e)) and outward (panel (f)) adaptation. Thin, low-opacity lines show the course of adaptation for all subjects. Thick lines on the top show the median value for all of the subjects for both paradigms in the two stimulation conditions. For the inward adaptation experiment no differences were observed in baseline or postadaptation phases but presented a significantly smaller gain under cerebellar tDCS condition (*P* = 0.02). In the outward adaptation experiment, subjects also presented a normal course of adaptation in which subjects in the sham condition present relatively smaller gains compared to tDCS condition observed since the baseline phase, though this was not significant in any of the three phases (see Section 3). Gray bars show the measures taken into account for the analysis in this study. (atDCS: anodal transcranial direct current stimulation. Post-A: postadaptation. Blue: sham, red: tDCS.)

The inward and outward adaptation experiments were created using Experiment Builder (SR Research, Ontario, Canada). In both experiments, the subject was instructed to look at a red dot (0.5 degrees of visual angle) displayed on a black background. At the beginning of the trial, the dot was shown at 10 degrees to the left of the center of the screen (fixation position). After a random delay between 1.5 s and 2 s, the fixation point was switched off and the dot appeared at a position on the right of the center (target position), evoking a visually guided saccade. In the inward adaptation experiment, this target position was 10 degrees to the right of the center and in the outward adaptation experiment the target position was 5 degrees to the right of the center. In other words, in the inward adaptation experiment the target jump was 20 degrees and in the outward adaptation experiment it was 15 degrees. Both experiments consisted of three phases with 250 trials in total (Figures 1(a) and 1(b)):

1. 50 baseline trials, where the dot remained on the rightward position for 1.5 seconds until the end of the trial;
2. 150 adaptation trials, in which the initial target position was the same; at saccade detection, however, the target jumped toward the fixation point in the inward adaptation experiment (i.e., backward target jump) and away from it (i.e., forward target jump) in the outward adaptation experiment during the saccade towards it; the size of the intrasaccadic step was 5° in both experiments; the saccade was detected online using a velocity threshold of 50°/sec and a boundary threshold of 7.5° to the right of the fixation position, to ensure that saccades were in the right direction; if no proper saccade was detected, the screen was blanked for 500 ms and the trial was presented again;
3. 50 “postadaptation” trials, identical to baseline trials.

### 2.4. tDCS

Anodal tDCS was delivered to the cerebellum through a constant current stimulator (NeuroConn, Ilmenau, Germany) through two annular sintered Ag/AgCl 12 mm diameter electrodes (MedCat, Erica, Netherlands) with highly conductive gel (Signa Gel, Parker Laboratories, New Jersey, USA) [32]. The anodal electrode was placed over the right cerebellum 3 cm to the right of the inion and the reference electrode (cathode) was placed over left buccinator muscle. The total current density was 1.3 mA/cm^2^, ramped up in 30 s to a constant 1.5 mA. Stimulation commenced 3 min before an experiment started and lasted for 15 minutes (i.e., during all baseline trials and adaptation trials). These criteria are well below the threshold for tissue damage [33–35].

### 2.5. Design

A subject participated twice in an experiment, once in a sham tDCS condition and once in an anodal cerebellar tDCS condition. The order of the tDCS conditions was pseudorandomized and counterbalanced across subjects, with three to seven days between recordings. In the anodal cerebellar condition, real stimulation was applied, while, in the sham condition, the current was turned off after 30 s [36]. Subjects and experimenter were blind to the tDCS condition (double-blind design). At the end of each paradigm, subjects were asked to report perceived pain and fatigue using a verbal analog scale (0—no fatigue/pain—to 5—maximal fatigue/pain), as well as the presence of headache, balance, nausea, and discomfort. Recordings in subjects who participated in both the inward and outward adaptation experiments were separated by at least seven days to avoid carry-over effects.

### 2.6. Data Analysis

For each trial, the primary saccade from the left (fixation) to the right (target) was analyzed. Saccades were marked automatically using a velocity threshold of 50°/s and a duration threshold of 20 ms. Trials were excluded if (1) there was no fixation inside a 1.7° window around the fixation point or (2) there was no saccadic movement from left to right. The amplitudes of the primary saccades were transformed into gain values, with gain being defined as the ratio between saccade amplitude and the distance between fixation and target position. A gain of 1 indicates a saccadic amplitude of 20° in the inward and 15° in the outward paradigm. The data was tested for normality using a Kolmogorov-Smirnov test. Median, mean, and SD of the gains were calculated for individual subjects and pooled by paradigm and condition. Saccades that fell outside ± 1.96 SD from the mean of a subject were excluded separately for every phase. From the inward adaptation experiment 4.76% of trials were excluded and 3.56% from the outward adaptation experiment.* Baseline gain* was defined as the median gain in all baseline trials,* adaptation gain* as the median gain of the last 10 saccades made in the adaptation phase, and* postadaptation gain* as the median gain of the last 10 saccades in the postadaptation phase.* Adaptation gain-change* was calculated as the difference between* adaptation gain* and* baseline gain*.* Retention* was calculated as the difference between the* postadaptation gain* and* baseline gain*, giving a measure of how much learning was retained after the adaptation phase.

Statistical analyses were performed using a custom script written in MATLAB (MathWorks, Natick, MA, USA), and SPSS (v. 20.0, IBM Corp., Armonk, NY, USA). We assessed the presence of adaptation for each subject by testing the difference between* baseline* and* adaptation* gain with a Student's* t*-test. For both inward and outward experiments, gains and saccade kinematics (duration and peak velocity) were analyzed using repeated measures' MANOVA with two intrasubject factors: tDCS condition (two levels: sham versus cerebellar tDCS) and phase (three levels: baseline, adaptation, and postadaptation). Post hoc planned comparisons between the two stimulation conditions for each of the three phases were performed using paired* t*-tests on the saccadic gains. The effects of tDCS on* adaptation gain-change* and on* retention* were assessed using paired* t*-tests.

For each experiment, the difference in* adaptation gain-change* and the difference in* retention* between tDCS and sham stimulation were calculated. These differences were statistically compared between the inward and outward experiments using a Wilcoxon signed rank test using the 7 subjects that participated in both experiments.

Pain and fatigue were statistically assessed using a one way ANOVA with tDCS condition as intrasubject factor. Statistical significances were set at *P* < 0.05.

## 3. Results 

All participants successfully completed the experiments and showed a significant change in gain during the adaptation phase. Example data of one subject and group data are shown in Figures 1(c) and 1(d), respectively.  Table 1 summarizes the results obtained in each phase for inward and outward adaptation for the two tDCS conditions. Pain and fatigue scores were not different between the tDCS or sham conditions (*P* > 0.5).

### 3.1. Inward Adaptation

A MANOVA on the gains for the inward adaptation experiment with sham and cerebellar tDCS and phase as factors revealed an effect of tDCS condition (*F*(1,9) = 6.755, *P* = 0.02, *η* ^2^ = 0.429) and phase (*F*(2,8) = 49.801, *P* ≤ 0.0001, *η* ^2^ = 0.926) as well as the interaction between tDCS condition and phase (*F*(2,8) = 6.439, *P* = 0.02, *η* ^2^ = 0.617; Table 1).

Saccades during baseline trials tended to be slightly hypometric for both the sham and tDCS conditions (Table 1), which is normal for saccades above 10 degrees [37, 38]. The adaptation phase showed a gradual decrease in gain throughout the trials in the two tDCS conditions, in which smaller gains are present for the tDCS condition (Table 1). In the postadaptation phase, we found that subjects in both groups did not present full recovery to baseline gains (Table 1).

Planned comparisons between the two conditions (sham and tDCS) showed no significant differences between the two stimulation conditions in baseline gains (0.95 ± 0.01 versus 0.96 ± 0.02, *t*(9) = 0.88,  *P* = 0.39, Figure 1(e)). The gain at the end of the adaptation phase was significantly smaller under cerebellar tDCS compared to sham stimulation (sham 0.83 ± 0.04, tDCS 0.81 ± 0.03, *t*(9) = −2.71, *P* = 0.02, Figure 1(e)). Postadaptation phase did not exhibit differences between the two conditions (sham 0.92 ± 0.03, tDCS 0.90 ± 0.03, *t*(9) = −1.75, *P* = 0.11, Figure 1(e)).

**Figure 2:**
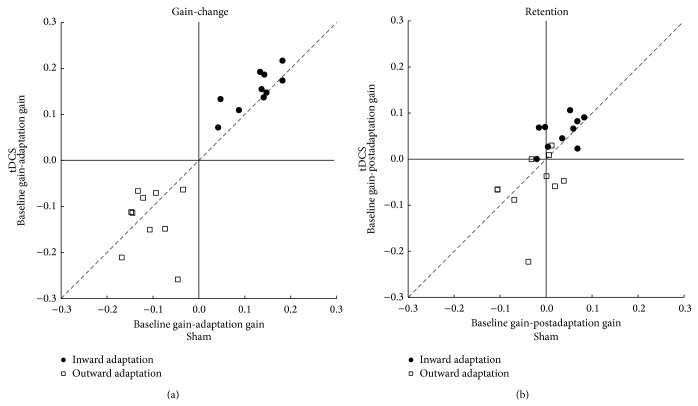
Adaptation gain-change and retention contrast between tDCS and sham condition. (a) Gain-change for inward and outward adaptation in which we can observe higher changes in gain (learning) for inward saccade adaptation with anodal cerebellar tDCS compared to sham stimulation. No difference is observed in outward saccade adaptation gain-change between anodal cerebellar tDCS and sham stimulation. (b) Retention (difference between baseline and postadaptation) for inward and outward adaptation in the two stimulation conditions.

The optimal adaptation gain-change (difference between baseline and the last 10 adaptation trials) is of 25% (gain of 1 to 0.75). The observed adaptation gain-change was lower in the cerebellar tDCS condition than in the sham condition (0.12 ± 0.04 versus 0.15 ± 0.03, *t*(9) = 2.26, *P* = 0.04, Figure 2). Difference in retention, which reveals learning residual between the two conditions, was just not significant (*t*(9) = 2.09, *P* = 0.06, Figure 2).

**Figure 3:**
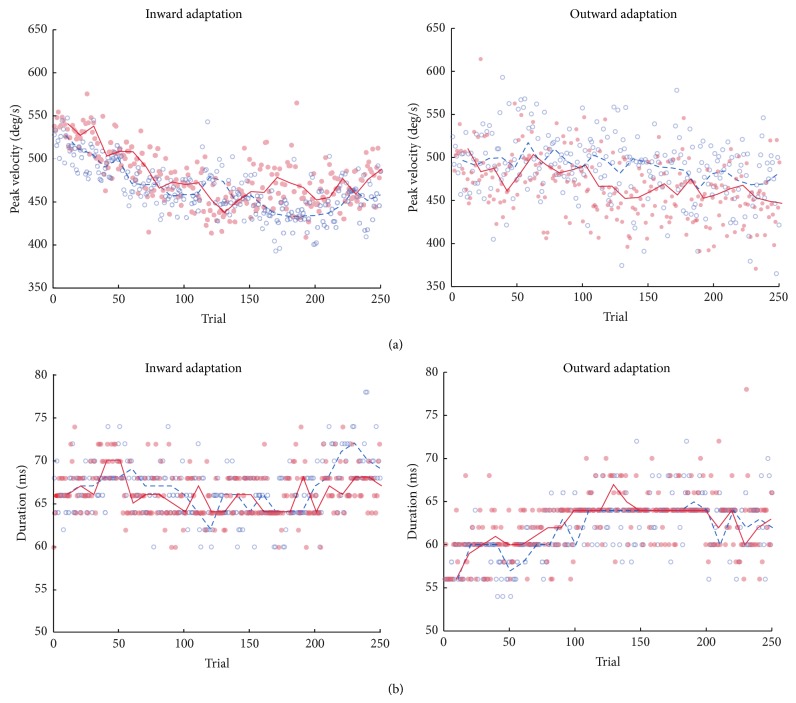
Saccade kinematics for sham and tDCS conditions. Left panel shows inward adaptation experiment and right outward adaptation experiment. (a) Peak velocity evolution throughout the trials as median values for all the subjects. Line on the top depicts blocks composed of the median values of 10 trials. For inward adaptation (left) a clear reduction of the velocity is observed as gains become smaller, not present in the same way for the increasing gains in outward adaptation (right). (b) Saccade durations as median values for all subjects. Line on the top depicts blocks composed of the median values of 10 trials. On the left, saccade durations become slightly smaller as gains become smaller. On the right, saccade durations increase as the task evolves as a result of saccade lengthening.

We also assessed if differences in saccade kinematics were present. Repeated measures' MANOVA analyses revealed an effect of phase on peak velocities (baseline: 518 ± 21 deg/s, adaptation: 452 ± 22 deg/s, and postadaptation: 499 ± 29 deg/s, *F*(2,8) = 17.45, *P* = 0.001, *η* ^2^ = 0.814), but the effects of tDCS condition (*F*(1,9) = 1.00, *P* = 0.34, *η* ^2^ = 0.101) or the interaction between tDCS condition and phase were not significant (*F*(2,8) = 1.24, *P* = 0.33, *η* ^2^ = 0.23; Figure 3(a)). No significant effects were found for saccade durations (Figure 3(b)).

### 3.2. Outward Adaptation

Here, participants were subjected to an outward intrasaccadic jump of the target in the adaptation phase. As in inward adaptation, subjects received anodal stimulation during baseline and adaptation phases (Figures 1(a) and 1(b)).  Figure 1(d) shows an example subject during the outward adaptation experiment in the two conditions. The resulting data from all subjects was approached in the same way as the previous experiment. The MANOVA analyses presented a main effect of phase on saccadic gains (*F*(2,8) = 51.10, *P* ≤ 0.0001, *η* ^2^ = 0.927) and on the tDCS condition (*F*(1,9) = 8.36, *P* = 0.01, *η* ^2^ = 0.482), whereas the tDCS condition and phase interaction was not significant (*F*(2,8) = 0.658, *P* = 0.544, *η* ^2^ = 0.141; Table 1).

Planned comparisons did not show any statistical difference between the two tDCS conditions. When observing group data during baseline, subjects present relatively smaller gains in sham condition compared to tDCS (sham 0.98 ± 0.03, tDCS 1 ± 0.02, *t*(9) = 1.79, *P* = 0.10). Subjects also presented a normal course of adaptation throughout the trials increasing their gains (sham 1.08 ± 0.04, tDCS 1.12 ± 0.07, *t*(9) = 1.97, *P* = 0.08), to thereafter decrease them in the postadaptation phase (sham 1 ± 0.06, tDCS 1.05 ± 0.07, *t*(9) = 2.23, *P* = 0.05), not reaching baseline again (Figure 1(f)).

Here we also assessed the amount of learning (gain-change) of each individual by comparing the baseline and the last 10 trials of the adaptation phase. The ideal amount of change in adaptation was 0.25, from 1 to 1.25. No significant differences were found between sham and tDCS conditions (*t*(9) = 0.79, *P* = 0.44) or for retention (*t*(9) = −1.21, *P* = 0.25, Figure 2).

Kinematic differences were assessed in the same way as gains: no effects of tDCS condition or the interaction between phase and tDCS condition, except for an effect of phase on saccade durations as a result of gain increase (baseline 58 ± 1 ms, adaptation 67 ± 4 ms, and postadaptation 63 ± 1 ms, *F*(2,8) = 21.89, *P* = 0.001, *η* ^2^ = 0.84; Figure 3).

### 3.3. Comparison between Inward and Outward Adaptation

Inward and outward adaptation did not differ from each other in the seven subjects that participated in both experiments with respect to adaptation gain-change (0.16 ± 0.03 versus 0.11 ± 0.07, Wilcoxon *Z* = −1.35, *P* = 0.17) or retention (0.05 ± 0.03 versus 0.06 ± 0.07, Wilcoxon *Z* = −0.33, *P* = 0.73).

## 4. Discussion

We observed that applying tDCS with a small contact electrode at 1.5 mA in an inward saccade adaptation experiment, with a 25% backward intrasaccadic step, induces a greater gain reduction when compared to sham condition. The effect of tDCS on gain-change is just not significant for outward adaptation, probably due to the low number of subjects.

Saccade adaptation is a widely used model for motor learning. When the eyes land on a location where a target was displayed, the oculomotor system detects that an error has been made and updates its motor commands to adjust its amplitude on a trial-by-trial basis. We have explored the use of tDCS in a different type of motor learning in which previous results from other research groups have demonstrated the effect of this type of stimulation on cerebellar output. Galea et al. [39] showed that anodal tDCS enhanced acquisition in a visuomotor transformation task by stimulation over the cerebellum and other experiments have also shown the effects of cerebellar tDCS in learning [30], attention [40], or working memory [28, 29]. The results of this study also show that tDCS exerts modulatory effects in behavior when applied to the cerebellum. The affirmation for the confined effects of the stimulation over the cerebellum is demonstrated mostly by previous reports of similar configurations which did not find any effects on brainstem or visual cortex [27, 41, 42] and the use of modeling techniques, [43, 44] in which the current flow has been proven uniform [45] with good sensitivity and response by Purkinje cells (PC) [46].

During the postadaptation the subjects must deadapt and any difference in this phase could indicate an effect on retention or a continuous effect of tDCS on (de)adaptation. While we did not see any significant difference the groups here, our sense is that this does not necessarily reflect a real lack of effect. Our sample size and the degree of noise here make strong conclusions difficult. In any case, this is not the main issue that this research sought to address. This finding is consistent with the work of Galea et al. [39], Jayaram et al. [30], and Zuchowski et al. [41] who observed differences in the speed of adaptation but found no poststimulation effects in the extinction rate of the learned response in their tDCS group.

There are some possible explanations for the lack of differences between the two conditions in outward adaptation. The mechanisms for these two types of adaptation are not completely understood and are thought to involve different neural substrates. Diverse theories explore why this could be happening, such as a natural tendency of the system to be hypometric, and this way reducing gains will develop in a faster way than increasing them [1]. A study by Liem et al. [18] using functional MRI showed that forward and backward error target shifts elicited different cerebellar activation patterns. Also, different behavioral mechanisms might be in place for the two types of adaptation, namely, a target remapping for outward adaptation [16].

Preliminary results by Panouilleres et al. [47] on saccade adaptation showed that anodal stimulation tended to slow down adaptation in both directions, while cathodal stimulation enhanced outward adaptation. Differences could arise on account of different electrode size, position, current, and time of stimulation as with other studies where apparent opposite effects might be present.

On inward adaptation, significant differences were found in peak velocities due to the gain-decrease adaptation and on outward adaptation we only observed a significant increase in the duration of the saccades as a result of adaptation. This suggests that tDCS is exerting an effect in the stages or at a level where saccade kinematics are not coded yet. This supports the notion that tDCS actually affects adaptation and not the saccade generation per se [8].

Direct comparison between the two paradigms yielded no significant results on the tDCS effects. Despite the fact that the effect sizes are almost similar for the two experiments, outward adaption presents larger noise in the resulting data. We presume that this increased noise does not prevent tDCS from having an effect on performance or learning, but it may still cause that the effect of tDCS on outward adaptation failed to reach significance. The current inability to stimulate specific areas in the cerebellar cortex could also account for the apparent lack of response in outward adaptation. Another probable source is a difference in the mechanisms needed to elicit either type of adaptation. In other words, we think a preliminary hypothesis that the effect exists in both inward and outward adaptation is a good starting point for further exploration. Total cerebellectomies abolish complete means of adaptation [21]; oculomotor vermis inactivation [48] impairs adaptation without affecting the production of saccades. Results from Kojima et al. [49] inactivated the same area with total incapacity for outward adaptation and a partial effect for inward adaptation. An MRI-guided TMS study [26] on Crus I had a dual effect on saccade adaptation, potentiating gain-up adaptation after effects and depressing gain-down adaptation. We suggest that tDCS might have enhanced the cerebellar plastic mechanisms needed for a more prominent participation of the cerebellum in inward adaptation.

Being able to modulate cerebellar output earns particular interest as PC change their firing pattern in response to saccade adaptation. As observed by Catz et al. [50] while recording PC activity in primates performing an inward and outward adaptation task, they observed a change in the population burst throughout the course of adaptation. The population signal may have a modulatory role throughout the saccade, which could in turn be modulated or broadened by applying tDCS [51]. This way, tDCS possibly elicits regional modifications to cerebellar output during saccade adaptation [27]. Extracellular recordings in primates have shown that inward adaptation increased PC complex spike activity [52]. Consequently, PC activity may be enhanced and more “sensitive” to error at the individual level; and at a regional level, tDCS might engage faster areas that are available for adaptation [30]. Assumptions of local and regional cerebellar stimulation are further supported by modeling studies [53] where somatic polarization together with axon terminal polarization seems to be key to the direct current response.

Another possible mechanism that tDCS could be possibly influencing is by affecting short-term plasticity through brain-derived neurotrophic factor (BDNF). BDNF is involved in synaptic plasticity and its secretion affects motor learning in humans [54]. TrkB, the receptor for BDNF, is located at the parallel fiber to PC synapse, where plasticity in the cerebellum takes place and might be regulating PC/parallel fiber mechanisms underlying short-term synaptic plasticity [55, 56]. Tests have shown that direct current stimulation plays a critical role in long-lasting synaptic potentiation in mouse slices [57]. At this moment, only inferences can be made of how tDCS might be working at a cellular level and more studies are needed in this area to elucidate what the actual effects of tDCS at the PC level are.

In conclusion, we showed an effect of tDCS over the cerebellum in an inward saccade adaptation task displayed by a greater gain-reduction compared to sham stimulation. We could not demonstrate a similar effect in the outward adaptation task, although we also could not rule one out. Moreover, we contribute to the evidence that cerebellar tDCS may be used to enhance cerebellar (oculomotor) function. TDCS could help lead the way to a better understanding of motor learning and how the cerebellum is contributing to each of these processes; therefore, more studies are needed to clarify the extent and the mechanisms through which tDCS can modulate cerebellar functions.

## Figures and Tables

**Table 1:** Saccadic gains, kinematics, adaptation gain-change, and retention. Gains, saccade kinematics (peak velocity and duration) measured during the three phases for inward and outward adaptation in the two conditions. Inward and outward adaptation gain-change for the two conditions shows the difference between preadaptation and adaptation phases. Adaptation phase values are the last ten trials (adaptation gain). Peak velocity in deg/s and Duration in ms.

Phase	Inward		Outward	
	Sham	tDCS	Sham	tDCS
Baseline				
Gain	0.95 ± 0.01	0.96 ± 0.01	0.98 ± 0.03	1 ± 0.02
Peak velocity (deg/s)	503.20 ± 79.02	534.70 ± 69.79	493.40 ± 104.81	479.15 ± 103.32
Duration (ms)	67.60 ± 8.93	68 ± 6.25	57.60 ± 4.69	60 ± 7.77
Adaptation				
Gain	0.83 ± 0.04^*^	0.81 ± 0.03^*^	1.08 ± 0.04	1.12 ± 0.07
Peak Velocity (deg/s)	450.85 ± 83.30	454.35 ± 87.42	492.35 ± 101.47	445.25 ± 104.76
Duration (ms)	67.20 ± 8.01	69 ± 16.68	65.60 ± 9.60	69.20 ± 15.52
Postadaptation				
Gain	0.92 ± 0.03	0.9 ± 0.03	1 ± 0.06	1.05 ± 0.07
Peak velocity (deg/s)	481.65 ± 128.86	517.90 ± 92.55	461.15 ± 131.26	490.50 ± 116.08
Duration (ms)	69 ± 8.70	65.80 ± 5.37	65 ± 10.55	61 ± 3.43
Adaptation gain-change	0.12 ± 0.04^†^	0.15 ± 0.03^†^	0.10 ± 0.04	0.12 ± 0.08
Retention	0.03 ± 0.03	0.05 ± 0.03	−0.02 ± 0.05	−0.04 ± 0.07

Values are mean ± SD. ^*^ *P* = 0.02, ^†^ *P* = 0.04.
